# Divide and Conquer-Based 1D CNN Human Activity Recognition Using Test Data Sharpening †

**DOI:** 10.3390/s18041055

**Published:** 2018-04-01

**Authors:** Heeryon Cho, Sang Min Yoon

**Affiliations:** HCI Lab., College of Computer Science, Kookmin University, 77, Jeongneung-ro, Seongbuk-gu, Seoul 02707, Korea; heeryon@kookmin.ac.kr

**Keywords:** human activity recognition, one-dimensional convolutional neural network, test data sharpening

## Abstract

Human Activity Recognition (HAR) aims to identify the actions performed by humans using signals collected from various sensors embedded in mobile devices. In recent years, deep learning techniques have further improved HAR performance on several benchmark datasets. In this paper, we propose one-dimensional Convolutional Neural Network (1D CNN) for HAR that employs a divide and conquer-based classifier learning coupled with test data sharpening. Our approach leverages a two-stage learning of multiple 1D CNN models; we first build a binary classifier for recognizing abstract activities, and then build two multi-class 1D CNN models for recognizing individual activities. We then introduce test data sharpening during prediction phase to further improve the activity recognition accuracy. While there have been numerous researches exploring the benefits of activity signal denoising for HAR, few researches have examined the effect of test data sharpening for HAR. We evaluate the effectiveness of our approach on two popular HAR benchmark datasets, and show that our approach outperforms both the two-stage 1D CNN-only method and other state of the art approaches.

## 1. Introduction

The proliferation of smartphones and other mobile devices have enabled real-time sensing of human activities through device embedded sensors such as accelerometers, gyroscopes, cameras, GPS, magnetometers, etc. Initially, one or more dedicated, standalone on-body sensors were attached to the specific parts of the human body for Human Activity Recognition (HAR) [1,2,3]. As smartphone usage became prevalent, HAR research shifted from using dedicated on-body sensors to exploiting smartphone embedded sensors for human activity data collection [4,5,6]. The activity recognition performance has greatly improved since the inception of HAR research, but the experimental set up varied among existing researches, for example, the types of activities performed by human subjects, the types of sensors employed, the signal sampling rates, the length of time series data segment, the feature processing techniques such as feature transformation, selection, extraction, the choice of classifier learning algorithms, and so on. These choices made comparative assessment of different HAR approaches difficult.

As HAR research matured, several benchmark human activity datasets [7,8,9,10,11] became publicly available, allowing straightforward comparison of different activity recognition methods. Recently, many state of the art approaches employ deep a Convolutional Neural Network (CNN) over other machine learning techniques, and these approaches, for example, exhibit high activity recognition accuracy that exceed 95% [12,13,14] on the benchmark Human Activity Recognition Using Smartphones Data Set (UCI HAR dataset) [10] that contain six activities. As deep learning approaches simultaneously learn both the suitable representations (i.e., features) and activity classifier from data, less attention was given to the explicit feature processing for HAR. Indeed, several existing works did exploit various feature processing techniques such as application of noise reduction filters to remove noise from human activity signals [10,15] while others transformed raw activity signals to frequency domain features using discrete Fourier transform [16] or discrete cosine transform [17]. However, few have investigated the effect of performing *data sharpening* for improving HAR. Moreover, the effect of data sharpening *on test data alone and not on the training data* was rarely examined.

**Figure 1:**
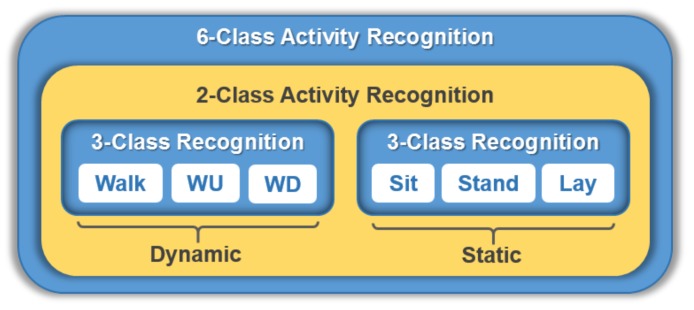
Division of 6-class HAR into two-stage *n*-class HAR. Six activities, i.e., Walk, WU (Walk Upstairs), WD (Walk Downstairs), Sit, Stand, and Lay, are divided into two groups of abstract activities, Dynamic and Static, to form a 2-class HAR. Each abstract activity forms a 3-class HAR.

In this paper, we present a novel one-dimensional (1D) CNN HAR that utilizes a divide and conquer-based classifier learning with test data sharpening for improving HAR. Suppose that we are faced with a 6-class HAR problem where the activities that need to be recognized are walking, walking upstairs (WD), walking downstairs (WD), sitting, standing, and laying, as shown in Figure 1. Instead of straightforwardly recognizing the individual activities using a single 6-class classifier, we apply a divide and conquer approach and build a two-stage activity recognition process, where abstract activities, i.e., dynamic and static activity, are first recognized using a 2-class or binary classifier, and then individual activities are recognized using two 3-class classifiers. During the prediction phase, we introduce test data sharpening in the middle of the two-stage activity recognition process to further improve activity recognition performance.

**Figure 2:**
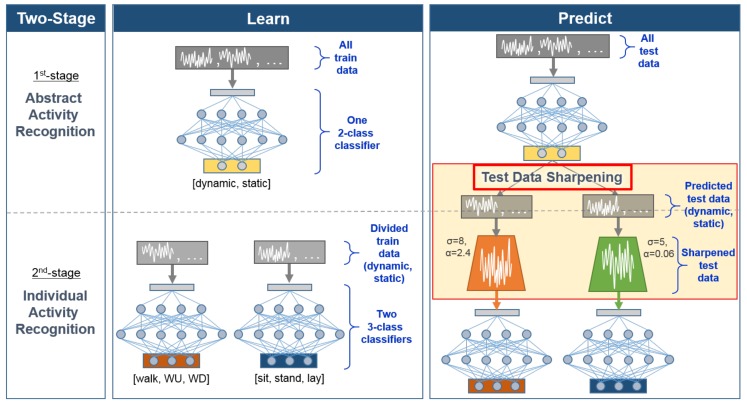
Overview of our divide and conquer-based 1D CNN HAR with test data sharpening. Our approach employs two-stage classifier learning during the learning phase and introduces test data sharpening during the prediction phase.

Figure 2 outlines the overall process of our divide and conquer-based 1D CNN HAR approach applied to Figure 1. The classifier learning is conducted via a two-stage process: in the first stage, a binary 1D CNN model for abstract activity recognition is learned for classifying dynamic and static activities; in the second stage, two 3-class 1D CNN models are learned for classifying individual activities. During the prediction phase, our method first classifies dynamic and static activity using the first-stage abstract activity recognition model, and then proceeds to test data sharpening. After the test data is sharpened, our approach inputs the sharpened test data into the relevant second-stage individual activity recognition model to output the final individual activity. The parameters (i.e., σ and α in Figure 2) required for appropriate test data sharpening is searched and selected using the validation data after the entire two-stage classifier learning process is completed and all three 1D CNN models are built. By breaking down the multi-class problem into simpler problem units and introducing test data sharpening in the prediction phase, we can achieve better HAR performance. We demonstrate the effectiveness of our approach using two benchmark HAR datasets, and show that our approach outperforms both the 1D CNN models without test data sharpening and existing state of the art approaches. The contributions of this paper are twofold:

1. We propose a divide and conquer approach for building two-stage HAR that incorporates test data sharpening during prediction phase to enhance HAR performance.
2. We present a systematic method for identifying useful parameters (i.e., σ and α) needed for test data sharpening.

The rest of the paper is structured as follows. The existing works on neural network-based HAR, two-stage HAR, and feature processing methods are reviewed in Section 2. The details of our divide and conquer-based 1D CNN approach with test data sharpening is presented in Section 3. The evaluation of our approach on two popular benchmark HAR datasets are reported in Section 4, and the experimental results are analyzed in Section 5. Finally, we conclude this paper in Section 6.

## 2. Related Work

In this section, we look at existing neural network-based HAR and other two-stage classifier learning techniques, and graze through some feature processing methods used in HAR along with signal sharpening in the image processing domain.

### 2.1. Neural Network-Based HAR

With the striking development in deep learning methods, many state of the art HAR algorithms now employ various deep neural network algorithms for recognizing human activities. Before deep learning was adopted, shallow neural network classifiers, namely Multi-Layer Perceptron (MLP), was utilized as a promising neural network algorithm for HAR. For example, et al. [18] compared decision tree, logistic regression, and MLP, and found that MLP triumphed in HAR. Dernbach et al. [19] compared MLP with naive Bayes and tree-based methods on simple (e.g., biking, climbing, stairs, driving, lying, running, sitting, standing, and walking) and complex (e.g., cleaning kitchen, cooking, medication, sweeping, washing hands, and watering plants) activity recognition, and found that MLP outperformed the rest. Bayat et al. [20] investigated the performance of MLP as both an individual classifier and as part of an ensemble classifier; as an individual classifier, MLP outperformed Support Vector Machine (SVM), random forest, logistic model trees, logistic regression, and additive logistic regression. Weiss and Lockhart [21] compared the relative performance of impersonal and personal activity recognition models using decision trees, k-nearest neighbor, naive Bayes, logistic regression, MLP, and so on, and found that MLP performed the best for personal models. Kwon et al. [22] investigated the influence of the smartphone triaxial accelerometer’s each axis on HAR using MLP.

Although MLP has shown competitive activity recognition performance, the algorithm is known to output poor recognition performance when it falls into local optima. Moreover, adding many hidden layers to MLP was difficult due to the vanishing gradient problem during back propagation [23]. To overcome such limitations of shallow classifier, deep neural network learning methods were introduced. For instance, Alsheikh et al. [24] implemented deep activity recognition models based on deep belief networks (DBNs). The first layer of their DBN consisted of Gaussian-binary Restricted Boltzman Machine (RBM), which modeled the energy content in continuous accelerometer data; the subsequent layers were modeled using binary-binary RBMs.

More recently, CNN-based algorithms have been applied to HAR for its advantages in capturing local dependency of activity signals and preserving feature scale invariance [25]. Existing works using CNN for HAR include [12,14,25,26]. Other approaches exploited deep Recurrent Neural Network (RNN) [27] or combined Long Short-Term Memory (LSTM) RNN with CNN. Ordonez and Roggen [28] proposed DeepConvLSTM that combined convolutional and recurrent layers. Here, abstract representations of input sensor data were extracted as feature maps in the convolutional layer, and the temporal dynamics of feature map activations were modeled in the recurrent layers. Edel and Köppe [29] proposed binarized bidirectional LSTM-RNNs that reduced memory consumption and replaced most of the arithmetic operations with bitwise operations achieving an increase in power-efficiency. In this study, we construct multiple 1D CNNs with various layer constitution.

### 2.2. Two-Stage HAR

Two-stage approaches break down a multi-class classification problem into several smaller multi-class problems. Sometimes they solve a complex activity classification problem using classification results of simple activities. In other cases, they utilize different subsets of sensor data at different steps of HAR. These two-stage approaches are interchangeably referred to as ‘two-level’, ‘two-step’, ‘two-phase’, ‘two-layer’ or ‘hierarchical’ approaches.

Many studies have introduced two-stage HAR. For example, Khan et al. [30] proposed a hierarchical activity recognition method that first distinguished the static, transition or dynamic state at the lower level, and then recognized more specific activities using linear-discriminant analysis and artificial neural network (ANN) at the higher level.

Lee and Cho [31] proposed a two-step Hierarchical Hidden Markov model (HMM) where a user’s *action* and *activity* were recognized sequentially. They defined user *action* as motion taken during shorter time lengths; five types of actions (i.e., stand, walk, stair up, stair down and run) were tested for action recognition. Once the user action was determined, a longer time window was set for recognizing user *activities*. Here, a series of recognized *actions* were fit into a longer time window as input for activity recognition. The user activities recognized were shopping, taking bus and moving by walking. The authors trained separate HMMs for action and activity recognition.

Widhalm et al. [32] proposed a two-stage classification technique that used a randomized ensemble of classifiers combined with a HMM in order to detect eight types of transport mode (i.e., bus, car, bike, tram, train, subway, walk, and motorcycle). They trained an ensemble of one hundred one-level decision trees with eight leaf nodes corresponding to the eight distinct modes of transport followed by a discrete HMM.

Han et al. [33] proposed a hierarchical activity recognition framework that first performed classification based on the current GPS data to see whether the GPS data matched one of the locations in the registered location list. If the GPS data matched one of the location in the registered location list, the user activity was recognized based on the GPS information alone or using other multimodal sensor data such as accelerometer and gyroscope data. If the GPS data did not match any of the location in the registered list, multimodal sensor data was used to identify user action. They used adaptive naive Bayes algorithm for activity recognition.

Hsu et al. [34] proposed a two-phase activity recognition using SVM to overcome the variance of smartphone sensor signals collected via different positions and orientations of the smartphone. In the first phase, the signals from the gyroscope were used to determine the position of the smartphone. Here, they defined three position types: the front pocket of the pants; the back pocket of the pants; and the shirt pocket, the backpack, the messenger bag, or the shoulder bag. Once the position type was recognized, the activity type was recognized in the second phase. They constructed three activity classifiers that corresponded to each of the three position types. For both phases of two-phase activity recognition, SVM was used.

Filios et al. [35] proposed a hierarchical activity detection model which consisted of a two-layer system that first detected motion and the surrounding environment (e.g., being in a coffee shop, restaurant, supermarket, moving car, etc.) using accelerometer data and microphone signals, and then detected more complex activities such as shopping, waiting in a queue, cleaning with a vacuum cleaner, washing dishes, watching TV, etc. based on the detected motion and environment information. They evaluated three decision tree-based algorithms and one *k*-nearest neighbor algorithm for recognizing motion, environment and complex activity.

Ronao and Cho [36] proposed a two-stage method that used two-level Continuous HMMs (CHMMs); the first-level CHMMs classified stationary and moving activities, and the second-level CHMMs classified more fine-grained activities, i.e., walking, walking upstairs, and walking downstairs for moving activities, and sitting, standing, and laying activities for stationary activities. They constructed a total of eight CHMMs, two CHMMs at the first-level and six CHMMs at the second-level, and chose different feature subsets when constructing different level CHMMs.

Our approach is similar to [36] in that we perform a two-stage classification where we classify abstract activities (e.g., dynamic and static) first and then classify individual activities (e.g., walking, standing, etc.) next. However, we build one binary 1D CNN model at the first stage and two multi-class 1D CNN models at the second stage. More importantly, we introduce test data sharpening in between the two-stage HAR, selectively at the prediction phase only, and this differentiates our approach from the rest of the two-stage HAR approaches.

### 2.3. Feature Processing

A typical activity recognition process comprises the stages for data acquisition, signal preprocessing and segmentation, feature extraction and selection, training, and classification [37]. The collected sensor signals usually contain noise that require filtering before being processed for HAR. Vision-based HAR systems also focused on feature extraction to remove noise from given data [38]. As a result, various preprocessing methods were introduced. For example, Khan et al. [30] incorporated a three-point moving average filter to remove signal outliers. Kozina et al. [15] used band-pass filter to eliminate both low-frequency acceleration that captured gravity component and high-frequency signals generated by noise. In contrast, Suarez et al. [39] utilized a low-pass filter to split the acceleration data into low- and a high-frequency components; these components were then used with the raw accelerometer data to increase activity recognition accuracy.

After the raw signals were processed and filtered, the filtered signals were often segmented using a fixed-size window, with or without the overlap of signals, to generate time series HAR data. From the segmented time series data, additional features were generated; signal characteristics such as time-domain and frequency-domain features were extensively utilized in HAR [40]. Such time-domain features include various statistical metrics such as mean, variance, standard deviation, etc., and envelope metrics such as median, maximum, minimum, range, etc. [41]. The frequency-domain features were computed using discrete Fourier transform (DFT) [16], discrete cosine transform (DCT) [17], discrete wavelet transform [42], spatio-temporal features [43], etc. Some frequency-domain features include peak frequency [41], power spectral density (PSD) [44], entropy [2], etc. A detailed overview of HAR preprocessing techniques is given in [41].

A typical HAR process usually applies feature processing on the entire dataset, i.e., both on the train and test data. To the best of our knowledge, there have been no research that have investigated the effect of feature processing *solely on the test data*. Most feature processing techniques have focused on the removal of noise and signal outliers from the activity signal or on the generation of time and frequency domain features. Almost no research, to our understanding, have applied test data *sharpening* during the prediction phase to improve activity recognition. In this respect, our approach of applying test data sharpening is novel and is worth the investigation.

With regard to signal sharpening, a technique called *unsharp masking*, which adds a high-pass filtered, scaled version of an image onto image itself has been frequently used in the image processing domain to improve visual appearance. Although this technique was seldom used in HAR, we investigate the effect of applying unsharp masking to activity signal. Many works on unsharp masking focus on enhancing the edge and detail of an image. For example, Polesel et al. [45] proposed an adaptive filter that controls the contribution of the sharpening path such that details are enhanced in high detail areas and little or no image sharpening occurs in smooth areas. Deng [46] proposed a generalized unsharp masking algorithm that allows users to adjust the two parameters to control the contrast and sharpness of the given image. Recently, Ye and Ma [47] proposed blurriness-guided adaptive unsharp masking method that incorporates the blurriness information into the enhancement process. Most works base their methods on the classic linear unsharp masking technique where image detail is extracted, amplified, and added back to the original image to produce an enhanced image. We follow this classic linear unsharp masking technique to sharpen activity signal in our approach.

## 3. Divide & Conquer-based 1D CNN HAR with Test Data Sharpening

As outlined in Figure 2, our approach conducts two-stage activity recognition by introducing test data sharpening in the middle of the two stages at prediction time. In this section, we first describe the identification of abstract activities required for first-stage HAR, and then explain methods for test data sharpening and selection of relevant sharpening parameter values.

### 3.1. Identifying Abstract Activity & Building 1st-Stage Classifier

At the outset, we tried to build a single sophisticated activity recognition classifier for multi-class HAR, but during the research process, we discovered that for certain pairwise activity classes, there were no misclassified instances. This finding could be easily visualized using an activity recognition confusion matrix.

**Figure 3:**
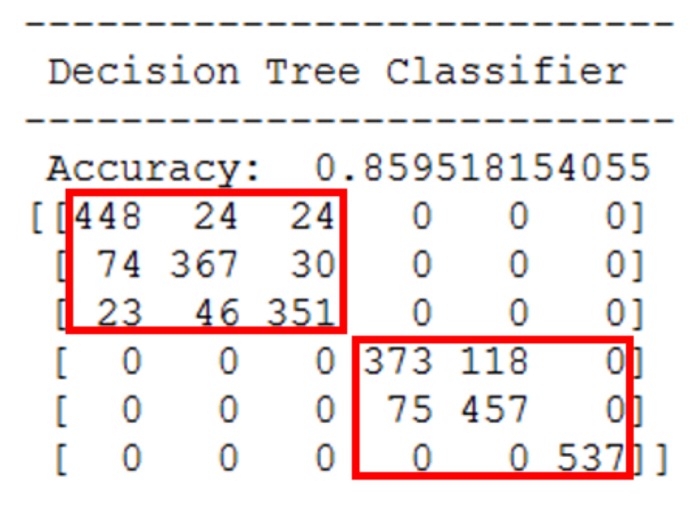
Confusion matrix of decision tree classifier on 6-class HAR. For some pairwise activity classes, there are no misclassified instances as indicated by the positions with zeros in the confusion matrix.

Figure 3 shows an example confusion matrix of decision tree classifier on 6-class HAR; the six activity classes are those given in Figure 1. The rows indicate the actual activity classes and the columns indicate the predicted classes. As shown, for some pairwise activity classes, there are no misclassified instances; i.e., the bottom left and the top right 3×3 submatrices all contain zeros. Meanwhile, the two red squares drawn over the confusion matrix contain both the correct and misclassified instances, and these demarcated activity classes can be transformed into abstract activity classes. The abstract activity classes are then utilized as target labels for building a binary classifier for first-stage HAR. In the case of Figure 3 confusion matrix, we converted walk, WD, and WU classes (Figure 3 top left) to dynamic class, and sit, stand, and lay classes (Figure 3 bottom right) to static class. It is important to note that recognition performance of first-stage binary classifier will determine the upper limit of the overall activity recognition accuracy since the second-stage activity classifiers, however perfect, will not affect the initial binary classification accuracy. While the divide and conquer approach is advantageous in that the complex (or many-class) classification problem is reduced to multiple simple (or less-than-many-class) classification problems, the approach requires that all classifiers reach reasonably good accuracy; a concerted effort among all simple classifiers are needed. Hence, whether to exploit the divide and conquer approach should be decided when at least the first-stage binary classification accuracy is reasonably high. In our experiments, the activity recognition accuracy of two first-stage binary classifiers on two benchmark datasets were 100%.

### 3.2. Building 2nd-Stage Classifier

Once we build a high-accuracy first-stage binary classifier, we proceed with the learning of the second-stage individual activity recognition classifiers. The detailed design and implementation of our second-stage 1D CNN models are described in Section 4. Once we finish building the best possible classifiers for the second-stage individual activity recognition, we move to test data sharpening.

### 3.3. Sharpening Test Data

**Figure 4:**
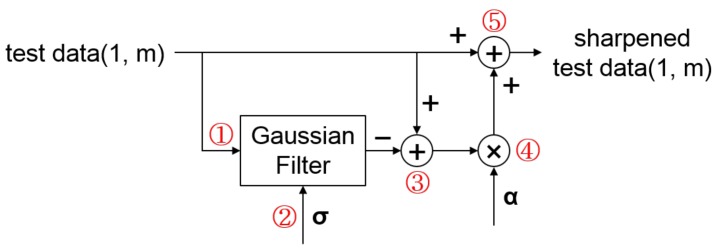
Test data sharpening using a Gaussian filter. Test data is first denoised using a Gaussian filter (①) using the σ parameter (②), and the denoised result is subtracted from the test data to obtain sharped details (③). The sharpened details are then amplified to some degree using α parameter (④) and added to the original test data to obtain sharpened test data (⑤).

Figure 4 shows the overall signal sharpening process applied to single test data. The train and test data for HAR are constructed in various formats, but usually they are formatted either as activity signal time series data in the form of matrices or as activity signal feature vectors. In the case of Figure 4, we assume that the test data is defined as an *m*-dimensional feature vector carrying various activity signal features. The test data sharpening is proceeded as follows: Firstly, a Gaussian filter is applied to the test data to remove minor features (see Figure 4① and Equation (1) below). The Gaussian filter has the effect of attenuating high frequency signals, and the degree of attenuation is determined by the σ parameter (Figure 4②). As a result, a denoised test data can be obtained. Next, the denoised vector is subtracted from the original test data vector to produce a fine detail vector (Figure 4③ and Equation (2)). The fine detail vector is then scaled by some scaling factor α (Figure 4④) before being added to the original test data vector (Figure 4⑤ and Equation (3)) to produce a sharpened test data.

(1) Denoised(1,m)=GaussianFilter(TestData(1,m),σ)

(2) Detailed(1,m)=TestData(1,m)−Denoised(1,m)

(3) Sharpened(1,m)=TestData(1,m)+α×Detailed(1,m)

**Figure 5:**
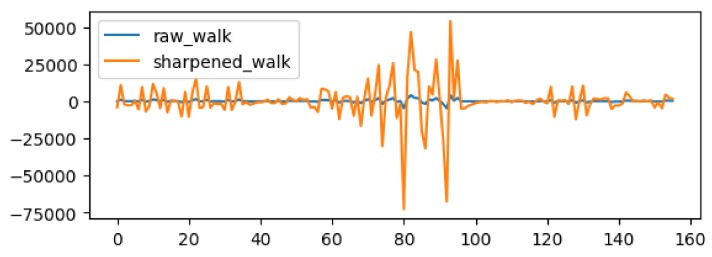
A sample activity data describing walking activity. Each number in the horizontal axis indicates various statistical features such as mean, standard deviation, minimum and maximum calculated from a fixed length time series data collected from multiple sensors. The blue line indicates data before sharpening and the orange line indicates data after sharpening.

Our idea of test data sharpening is borrowed from a popular signal enhancement technique called unsharp masking used in image processing for sharpening images. The visual appearance of an image may be improved significantly by emphasizing its high frequency contents to enhance the edge and detail of the image [45]. Often the classic linear unsharp masking technique is employed to enhance such details. The classic linear unsharp masking first generates a coarse image by removing the fine details from the image using a denoising filter, and then subtracts the coarse image from the original image to obtain fine details. Then the technique adds the fine details, often scaled by some factor first, to the original image to create a sharpened image. We have repurposed this unsharp masking technique to HAR domain and applied it to test data sharpening. Figure 5 shows a sample walking activity data before sharpening (blue line) and after sharpening (orange line).

### 3.4. Selecting Sigma & Alpha Values

**Figure 6:**
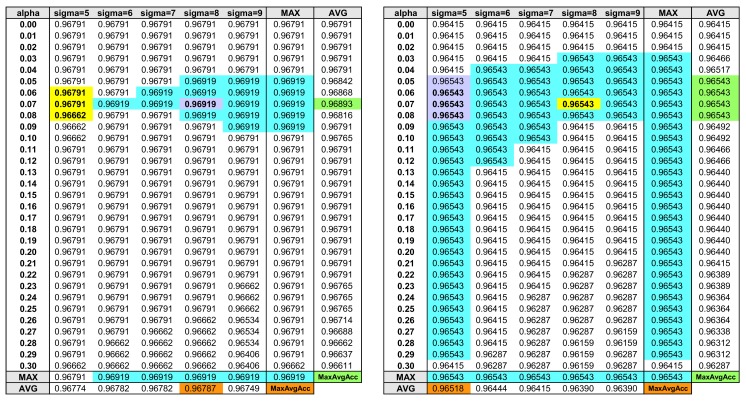
Validation/test data HAR accuracy using different (σ, α) combinations. The cyan colored cells indicate the highest activity recognition accuracy. Maximum of average accuracy (MaxAvgAcc) is searched for various average accuracies of σ and α values in order to find the suitable (σ, α) parameter values (orange and green colored cells). Assuming that the left table is the HAR accuracy of validation data, the purple cell where the two MaxAvgAccs meet identifies the suitable values (σ = 8, α = 0.07). Assuming that the right table is the HAR accuracy of test data, the yellow cell at (σ = 8, α = 0.07) achieves the highest accuracy of 96.543%.

As previously explained, the degree of test data sharpening is determined by the two parameters, σ (Figure 4②) and α (Figure 4④). Since the amount of test data sharpening affects the second-stage activity recognition accuracy, choosing a useful (σ, α) value combination is vital for successful second-stage HAR. Here, we explain a method for systematically selecting relevant parameter values for test data sharpening. Figure 6 displays two tables of second-stage activity recognition accuracy using different (σ, α) value combinations on validation and test data. The bottom two rows of each table indicate the maximum accuracy (indicated as MAX) and the average accuracy (AVG) using fixed σ values with varying α values, and the rightmost two columns of each table indicate the maximum accuracy (MAX) and the average accuracy (AVG) using a fixed α value with varying σ values. The cyan colored cells in the two tables indicate the highest activity recognition accuracy achieved among the various σ and α value (i.e., (σ, α)) combinations defined in the table. Notice that there exist multiple (σ, α) combinations that achieve the highest activity recognition accuracy. In order to choose a single (σ, α) combination among the numerous highest-accuracy value combinations, we find the *maximum of average accuracy* (MaxAvgAcc in Figure 6) among the various average accuracies of σ and α values. The orange and green colored cells at the bottom row and rightmost column of each table contain the MaxAvgAcc for σ and α values.

Once we find the MaxAvgAcc, we search the cell position(s) where the two MaxAvgAccs meet; this position is highlighted in purple in Figure 6. The purple cell in the left table has (σ=8, α=0.07), and the purple cells on the right table has (σ=5, α=[0.05,0.06,0.07,0.08]). The left table contains a final (σ=8, α=0.07) combinations for test data sharpening, but the right table contains multiple α values that need to be narrowed down further. If we still have multiple (σ, α) combinations as with the case of the right table, we calculate the average accuracy of the nearby accuracy triples either horizontally or vertically first and then on both sides (i.e., 3×3 cells with the target value combination positioned at the center).

Although test data sharpening is performed during prediction time, (σ, α) values for test data sharpening is determined using the validation data during the classifier learning phase after all two-stage classifiers are learned. Assuming that the left table in Figure 6 is the activity recognition result using the validation data, (σ=8, α=0.07) is determined as the final test data sharpening parameter values. If we suppose the right table to contain test data HAR performance with varying degrees of test data sharpening, using the (σ=8, α=0.07) pair achieves the highest activity recognition accuracy of 96.543% (see right table yellow cell). On the other hand, if we assume this time that the right table is the validation data and the left table is the result of sharpened test data, the three value combinations are selected (purple cells with bold font based on horizontal averaging of nearby triples), but when we apply any of these value combinations on test data sharpening (left table yellow cells), we obtain the test data accuracies of 96.791% or 96.662%. In this case, we fail to increase the baseline test data accuracy, which is given at the top row of left table where α=0 (refer to Equation (3)). The effectiveness of the test data enhancement depends on the representativeness of the validation data, but incorporating a broader range of σ and α values will help in choosing better parameter value combinations. In the case of the failed example just mentioned, if we expand our σ range to encompass 3 to 12, the candidate value combinations are reduced to (σ=5, α=[0.05,0.06,0.07]), and we can remove (σ=5, α=0.08), which leads to worse accuracy.

## 4. Evaluation Experiments

We used two public HAR datasets [8,10] in the evaluation experiments to compare our approach to other state of the art approaches and to our 1D CNN with no test data sharpening approach.

### 4.1. Benchmark Datasets

#### 4.1.1. OPPORTUNITY Activity Recognition Dataset

The OPPORTUNITY Activity Recognition Dataset (OPPORTUNITY dataset) [8] (https://archive.ics.uci.edu/ml/datasets/opportunity+activity+recognition) comprises body movement signals of four human subjects collected at 30Hz using body-worn sensors attached to various positions of the body. Each human subject performed five data recording sessions where they conducted naturalistic activities of daily living (ADL) and one drill session where they conducted a predefined set of activities instructed by the data collectors. The collected activity classes consisted of four basic activities, i.e., *stand*, *sit*, *walk*, and *lie*, and seventeen mid-level gestures such as *opening and closing of the fridge*, etc. In our experiment, we focused on recognizing the four basic activities.

We used the same test data employed in the OPPORTUNITY challenge as described in [48] (Task A: multimodal activity recognition: modes of locomotion) to evaluate our approach, but used smaller train data and larger validation data to train the model and to select the test data sharpening parameters. Note that we did not use any of the drill session data since the drill data contained artificially staged activity data. As a result, we trained our 1D CNN models using ADL1, ADL2, and ADL3 for Subject 1 and ADL1 and ADL2 for Subjects 2 and 3; we validated our model using ADL4 and ADL5 for Subject 1 and ADL3 for Subjects 2 and 3; we tested our model on ADL4 and ADL5 for Subjects 2 and 3. Following the OPPORTUNITY challenge’s experimental setup, we set the length of the sliding window as 500 ms with a step size of 250 ms. Each data contained 15 sample recordings. Prior to sampling, we grouped data according to same activity class labels so that no transition of activities were present in any of the sample recordings.

**Figure 7:**
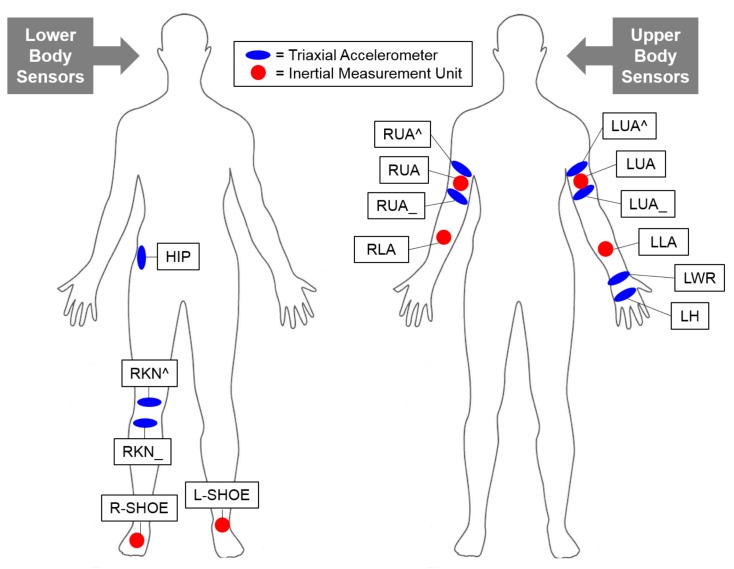
Lower (left) and upper (right) body sensors selected for OPPORTUNITY dataset experiment. For the lower body sensors, we chose three triaxial accelerometers (marked in blue) located at the right hip (HIP), right knee (RKN^), and right knee (RKN_), and three inertial measurement units (marked in red) located at the right (R-SHOE) and left shoe (L-SHOE) for the experiments. For the upper body sensors, we chose six triaxial accelerometers located at the right upper arm (RUA^), right upper arm (RUA_), left upper arm (LUA^), left upper arm (LUA_), left wrist (LWR), and left hand (LH), and four inertial measurement units located at the right upper arm (RUA), right lower arm (RLA), left upper arm (LUA), and left lower arm (LLA).

Although the OPPORTUNITY dataset contained rich sensor data collected using a total of nineteen sensors, we divided the sensors into lower and upper body sensors, and experimented with these two groups sensors. For the sensor data located at the lower part of human body, we selected three triaxial accelerometer placed at the upper (RKN^) and lower (RKN_) right knee and the right hip (HIP), and two inertial measurement units placed at the right shoe (R-SHOE) and left shoe (L-SHOE) as illustrated in Figure 7 (left). For the sensor data located at the upper part of human body, we selected six triaxial accelerometers located at the right upper arm (RUA^), right upper arm (RUA_), left upper arm (LUA^), left upper arm (LUA_), left wrist (LWR), and left hand (LH), and four inertial measurement units located at the right upper arm (RUA), right lower arm (RLA), left upper arm (LUA), and left lower arm (LLA) as illustrated in Figure 7 (right). The OPPORTUNITY dataset also included sensor data from the right wrist (RWR) and right hand (RH), but we did not include these two sensor data as upper body sensor data due to large number of missing values. We compared the HAR performance of upper and lower body sensors on the four basic activities, but we conjectured that the lower body sensors would yield better performance since the activities we need to predict were more influenced by the lower body movement. For both types of sensor data, we computed the mean, standard deviation, maximum, and minimum values of each of the 15 sample recordings and used these four measurements as the activity features instead of the raw 15-sample time series data. As a result, a total of 156 and 216 features were defined for lower and upper body sensor data respectively. The triaxial accelerometer records movement in the *x*, *y*, and *z* axes, so there were a total of nine measurements for the three accelerometers that we selected for the lower body sensors. Also, we used 15 measurements (excluding the compass) for each of the inertial measurement units, so the two inertial measurement units on the right and left shoe consisted a total of 30 measurements. Adding these together, there were 39 measurements. Instead of using the 15 sample recordings for each measurement, we used the four statistical measurements that we selected; consequently, 39×4=156. Similar calculations were performed for the upper body sensors to yield 216 features. Table 1 summarizes the class label constitution of the OPPORTUNITY dataset experiment. The same class label constitution applies to both the lower and upper body sensor data. Notice that we have introduced two posture classes, *up* and *down*, as abstract classes for the divide and conquer approach; these abstract classes indicate whether a person is in a raised position or in a lowered position. Using the activity class constitution in Table 1, we first learned a binary first-stage classifier that recognized abstract activities and then learned two binary classifiers that recognized individual activities.

#### 4.1.2. UCI HAR Dataset

The UCI HAR dataset [10] (https://archive.ics.uci.edu/ml/datasets/human+activity+recognition+using+smartphones) contains sensor signal recordings of thirty human subjects performing six activities, i.e., *walking*, *walking upstairs (WU)*, *walking downstairs (WD)*, *sitting*, *standing*, and *laying*, while carrying a waist-mounted smartphone with embedded inertial sensors. Using the smartphone’s embedded accelerometer and gyroscope, the triaxial linear acceleration and triaxial angular velocity were collected at 50 Hz. The collected signals were preprocessed by applying noise filters and then sampled in fixed-width sliding windows of 2.56 s and 50% overlap which resulted in 128 readings per window. A 561-feature vector consisting of time and frequency domain variables, which compute various measurements such as mean, standard deviation, minimum, maximum, etc. of the 128 readings of each sensor’s *x*, *y*, and *z* axes, were provided. We used all 561 features in the dataset and used the given train and test split to evaluate our approach. 20% of the train data was randomly selected as validation data. Table 2 summarizes the class label constitution of the UCI HAR dataset.

Here, we also split the dataset into two abstract activities, i.e., *dynamic* and *static*. Using the class constitution in Table 2, we hierarchically constructed one binary classifier for recognizing abstract activities and two 3-class classifiers for recognizing individual activities.

### 4.2. 1D CNNs: Design, Method, & Network Parameters

A total of five 1D CNN models, three for OPPORTUNITY dataset and two for UCI HAR dataset were constructed using open source machine learning software libraries (TensorFlow: https://www.tensorflow.org/, Keras: https://keras.io/) in the experiments.

#### 4.2.1. OPPORTUNITY Models

**Figure 8:**
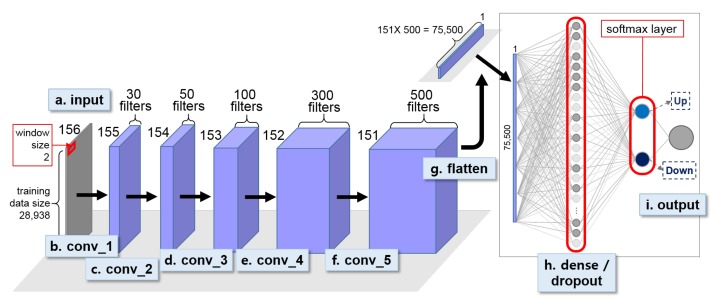
First-stage 1D CNN for classifying abstract activities, i.e., Up and Down, for OPPORTUNITY dataset.

For the OPPORTUNITY dataset experiment, we constructed one binary 1D CNN classifier for the first stage HAR and two binary 1D CNN classifiers for the second-stage HAR. The two second stage models were identical in design and network parameter configurations. We first constructed the first-stage model by stacking five consecutive convolutional layers (Figure 8) followed by a fully-connected, i.e., a dense layer with a 60% dropout rate and a softmax layer. A single window size of 2 and stride size of 1 was set to perform convolutions in all convolutional layers. We introduced early-stopping to prevent overfitting of the first-stage model.

**Figure 9:**
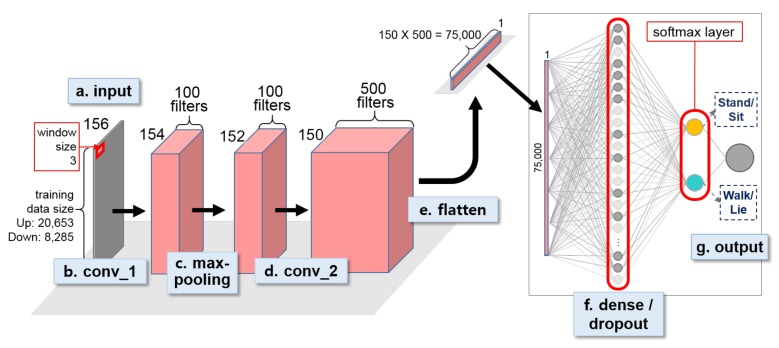
Second-stage 1D CNN for classifying individual activities for OPPORTUNITY dataset. Two identically designed 1D CNNs were constructed to distinguish Stand from Walk and Sit from Lie.

For the two identical second-stage models (Figure 9), one convolutional layer was followed by a max-pooling layer, followed by a second convolutional layer. A window size of 3 and stride size of 1 were applied to both convolution and max-pooling layers. Like the first stage model, a dense layer was positioned at the end of the network followed by the softmax layer, and 33% dropout rate was applied to the dense layer. The epoch and training batch size of the three models were set identically as 5 and 32 respectively. For all three models, Rectified Linear Unit (ReLU) was chosen as the activation function for all convolutional layers. The Mean Squared Error (MSE) was chosen as the loss function and the Adaptive Moment Estimation (ADAM) optimizer [49] was used in optimization for all models. The learning rate of the optimizer was set at 0.00006 and 0.00001 for the first- and second-stage model respectively.

#### 4.2.2. UCI HAR Models

**Figure 10:**
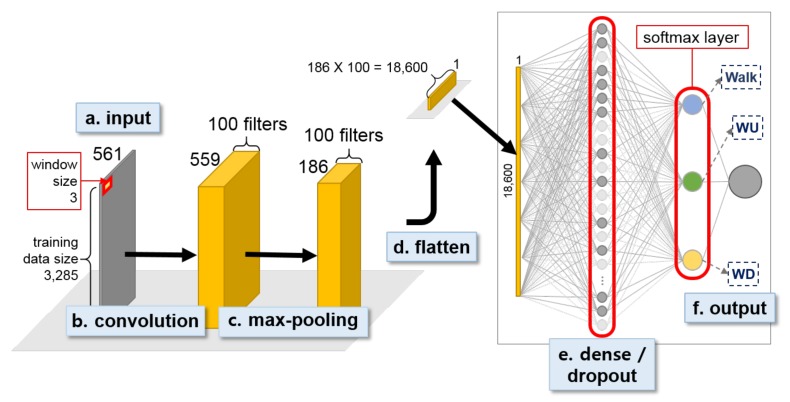
Second-stage 1D CNN for classifying dynamic activity, i.e., Walk, WU, and WD, for UCI HAR dataset.

**Figure 11:**
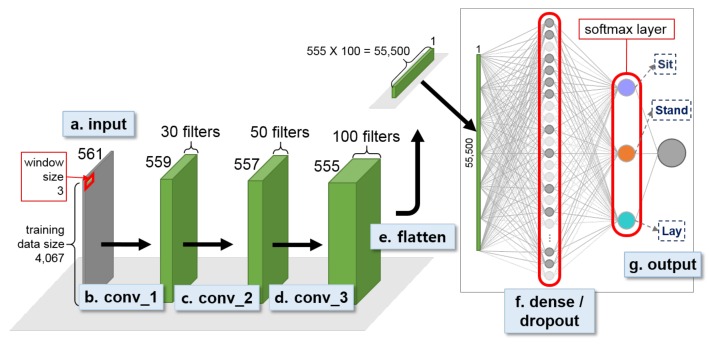
Second-stage 1D CNN for classifying static acitivity, i.e., Sit, Stand, and Lay, for UCI HAR dataset.

For the UCI HAR dataset experiment, we first built a first-stage binary decision tree classifier for recognizing dynamic and static activites, which achieved 100% activity recognition accuracy. An open source machine learning software library, scikit-learn (http://scikit-learn.org), was used to construct the decision tree classifier [50]. We then built two 3-class 1D CNN models for the second-stage individual activity recognition (Figure 10 and Figure 11).

Figure 10 and Figure 11 show the two second-stage 3-class 1D CNNs built using UCI HAR dataset. Both models used a sliding window size of 3 for convolution. While the dynamic activity model used max-pooling after convolution, the static activity model used three consecutive convolutions and no max-pooling. The max-pooling stride size was set equal to the window size of the convolution for the dynamic model. Both the dynamic and static models included a dense layer with 50% dropout rate and a softmax layer at the end of the network. We set the epoch size at 50 and 100 for the dynamic and static activity models respectively and saved the best models based on the validation loss. The MSE was chosen as the loss function, ADAM optimizer was used for optimization, the training batch size was set at 32 samples for all models, and ReLU was chosen as the activation function in all convolutional layers. The learning rate of the optimizer was set at 0.0004 and 0.0001 for the dynamic and static model respectively.

### 4.3. Test Data Sharpening

As explained in Section 3.4, the selection of σ and α values for test data sharpening was performed using the validation data. As the range and intervals of σ and α values affected the final (σ, α) value combination, we tested with various value ranges and intervals using the validation data. Two things must be noted: firstly, because the validation accuracy of both dynamic and static activity models for UCI HAR dataset were close to 100%, it was difficult to select (σ, α) values since the validation accuracy using the various parameter combinations resulted in either no improvement or deterioration. To remedy this situation, we exploited dynamic and static test data in the UCI HAR dataset by splitting the respective test data in half, and used the first-half of the test data as the validation data to select the (σ, α) values for the second-half of the test data and vice versa. Figure 6 in Section 3.4 is the actual result of the parameter selection using the static activity data in the UCI HAR dataset; the left table in Figure 6 corresponds to the result using the first-half of static test data and the right table corresponds to the result of the second-half of static test data.

Secondly, for the OPPORTUNITY dataset, for both the lower and upper body sensor data, we only performed test data sharpening on the ‘up’ activity recognition process, i.e., the classification of *stand* and *walk*, since the ‘down’ activity (i.e., *sit* and *lie*) recognition process already achieved 100% recognition accuracy using the raw, unsharpened test data. Table 3 displays the final (σ, α) combinations used in the experiments. For applying Gaussian filtering for test data sharpening, we used an open source multi-dimensional image processing software package. (https://docs.scipy.org/doc/scipy-0.19.1/reference/generated/scipy.ndimage.gaussian_filter.html#scipy.ndimage.gaussian_filter)

### 4.4. Baseline Methods & Evaluation Metric

We first compare our method’s HAR performance with the following state of the art approaches: using the lower body OPPORTUNITY dataset, we compare our approach to deep convolutional LSTM networks proposed by Ordónez and Roggen [28]; using the UCI HAR dataset, we compare our approach to four other approaches, i.e., one using SVM [10] (UCI HAR dataset owner), one using a two-dimensional activity image-based DCNN (DCNN+) [12], one using a fast Fourier transform-based DCNN (FFT+Convnet) [14], and one using a Three-Stage Continuous HMM (TSCHMM) [13]. We refer to our 1D CNN model with test data sharpening as CNN+Sharpen in the results section. In the case of OPPORTUNITY dataset, we additionally present the result of upper body sensor data, the effect of test data sharpening on end-to-end (i.e., 4-class) 1D CNN model using lower body data, and the result of test data sharpening on other machine learning techniques such as logistic regression and random forest. We also compare the performance between lower and upper body sensor data, and between raw time series data and statistical feature data using the lower body data. Finally, we compare our test data sharpening approach with the initial approach that does not use test data sharpening. We use activity recognition accuracy and $F_{1}$ score as evaluation measures. The confusion matrices of our results are provided for those cases where the results are compared to the existing approaches.

### 4.5. Performance Comparison

#### 4.5.1. OPPORTUNITY Dataset Result

Table 4 compares the activity recognition $F_{1}$ score (defined in [28]) of our approach using lower body OPPORTUNITY dataset with Ordónez and Roggen [28]’s approach. We see that our approach of 94.2% written in bold outperforms Ordónez and Roggen [28]’s. Table 5 shows the confusion matrix of our approach; note that test data sharpening was not employed in the ‘down’ activity (i.e., *sit* and *lie*) recognition since the default 1D CNN model correctly classified all test data. The overall accuracy was 94.27%.

**Figure 12:**
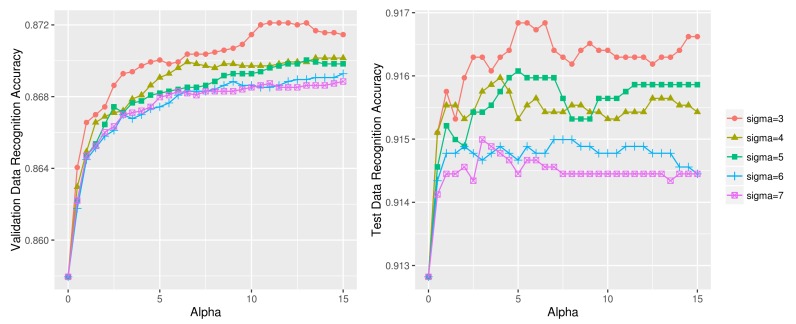
*Stand* vs. *walk* recognition accuracy using different (σ, α) combinations on lower body OPPORTUNITY dataset (**left**: validation data, **right**: test data).

Figure 12 compares the ‘up’ activity (i.e., *stand* and *walk*) recognition using different (σ, α) values on the lower body validation (left) and test data (right). The plot where α=0 indicates the activity recognition accuracy with no test data sharpening. We see that for both the validation and test data, all (σ, α) combinations given in the two graphs outperform the pre-test data sharpening model (α=0). Moreover, the relative position of the different line graphs having different σ values exhibit similar relative positions in the two graphs.

**Figure 13:**
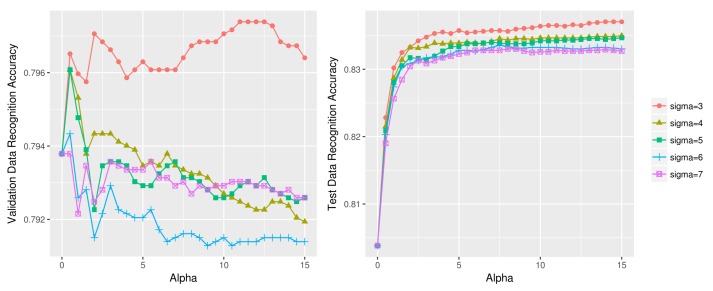
*Stand* vs. *walk* recognition accuracy using different (σ, α) combinations on upper body OPPORTUNITY dataset (**left**: validation data, **right**: test data).

**Figure 14:**
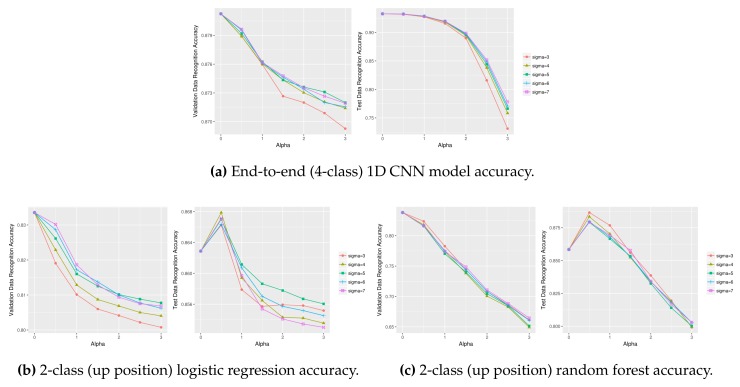
Unsuccessful test data sharpening cases using lower body data (left: validation, right: test).

Figure 13 compares the ‘up’ activity (i.e., *stand* and *walk*) recognition using different (σ, α) values on the upper body validation (left) and test data (right). The test data sharpening parameter values were determined as (σ=3, α=12) using the validation data, and the final accuracy of test data was determined as 83.66%; this accuracy is an improvement from the initial 80.38% with no test data sharpening. The 2-class accuracy of upper body sensor data (Figure 13), however, is much lower than the lower body sensor data of 91.63% (Figure 12). Figure 14 displays three unsuccessful test data sharpening cases. Test data sharpening is not effective for 4-class 1D CNN models (Figure 14a) and other machine learning techniques such as logistic regression (Figure 14b) and random forest (Figure 14c).

**Figure 15:**
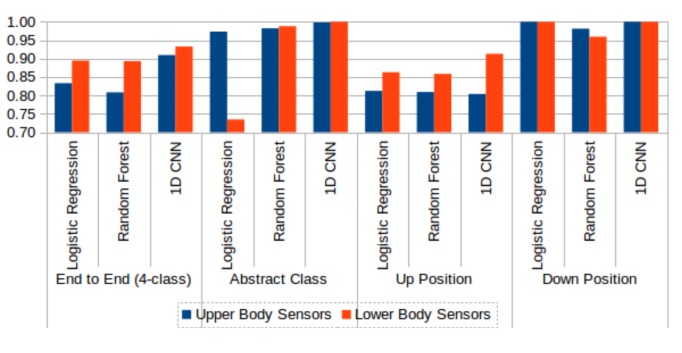
Comparison of upper body (blue) and lower body (red) sensor data performance without test data sharpening. Three machine learning techniques, logistic regression, random forest, and 1D CNN, are compared on 4-class and various 2-class problems.

**Figure 16:**
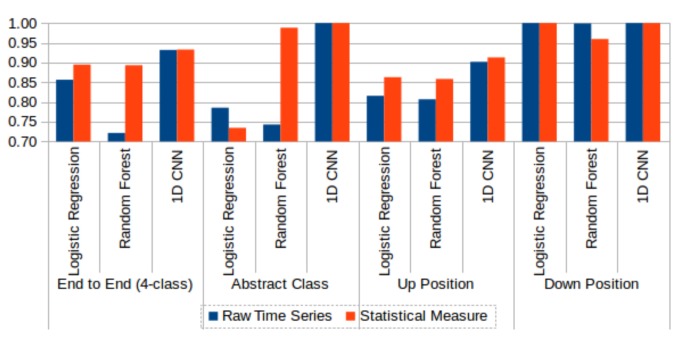
Comparison of raw time series (blue) and statistical feature (red) data performance without test data sharpening. Three machine learning classifiers, logistic regression, random forest, and 1D CNN, are compared on 4-class and various 2-class problems.

We performed additional experiments that compared various models constructed using upper and lower body sensor data. Figure 15 compares the two sensor types’ performances on 4-class and 2-class HAR problems. Overall, lower body sensor data (red bars) returned better results that upper body sensor data (blue bars). We also compared the performance of various models built using raw time series data and statistical feature data. Figure 16 compares raw time series data with statistical feature data using three machine learning classifiers, logistic regression, random forest, and 1D CNN. Overall, statistical feature data (red bars) returned better results than raw time series data (blue bars).

#### 4.5.2. UCI HAR Dataset Result

**Figure 17:**
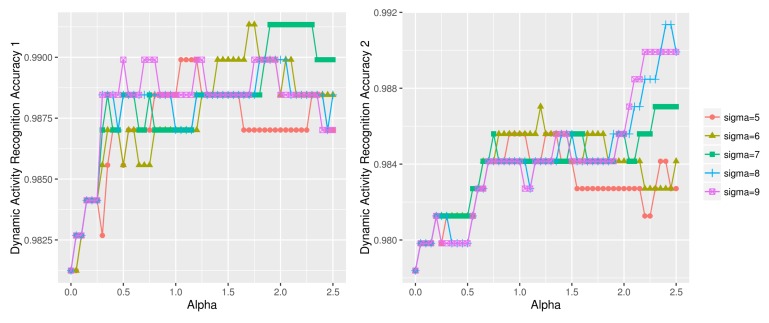
Dynamic activity recognition accuracy using various (σ, α) combinations on UCI HAR dataset (left: dynamic test data1, right: dynamic test data2).

**Figure 18:**
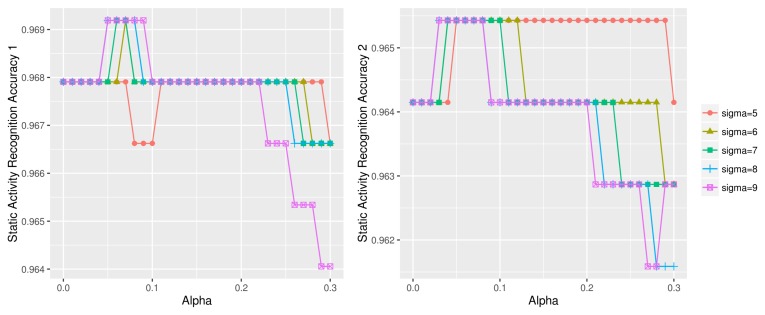
Static activity recognition accuracy using various (σ, α) combinations on UCI HAR dataset (left: static test data1, right: static test data2).

Table 6 compares activity recognition accuracy of our approach against other state of the art approaches using UCI HAR dataset. We see that our approach of 97.62% accuracy written in bold outperforms all other approaches. Table 7 shows a confusion matrix of our approach. We see that dynamic activity recognition model performs better than the static activity recognition model. The differentiation of *sit* and *stand* classes was the most difficult as evidenced by the relatively high number of the misclassified instances (14 and 37). Figure 17 and Figure 18 compare the dynamic and static activity recognition accuracy using various (σ, α) values. The final accuracy obtained from each graph was: 98.84% (Figure 17 left), 98.56% (Figure 17 right), 96.79% (Figure 18 left), and 96.54% (Figure 18 right).

#### 4.5.3. With & Without Test Data Sharpening

Table 8 compares the activity recognition performance within our approaches where test data sharpening was not performed (1D CNN only) and was performed (1D CNN+Sharpen). We see that test data sharpening works favorably in all three cases albeit with different rates of improvement. The increase in accuracy was more observed in models containing more active movements (i.e., Static < Up < Dynamic).

## 5. Discussion

### 5.1. Parameter Adjustment

The activity recognition accuracy of our approach ultimately depends on the two parameters for test data sharpening; Gaussian filter’s σ parameter, which adjusts the level of test data smoothing, and the scaling factor α used for controlling the level of fine detail, which is added to the original test data. Not enough smoothing of test data (e.g., low values such as σ=1,2) can lead to failure in extracting enough fine details in the subsequent step, and consequently, the activity recognition accuracy may worsen after test data sharpening.

**Figure 19:**
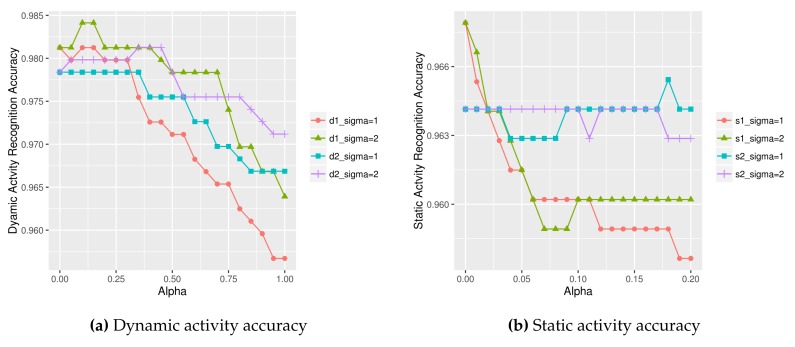
Dynamic (**a**) and static (**b**) activity recognition result using σ=1,2 on UCI HAR dataset.

Figure 19 illustrates this worsening effect on the UCI HAR dataset; the dynamic (left) and static (right) activity recognition accuracy decreases when test data sharpening is applied with σ=1,2. Note that ‘d1_sigma=1’ in Figure 19a, for example, indicates the activity recognition accuracy of the first half of the dynamic test data using σ=1. The plots at α=0 indicate the baseline accuracy with no test data sharpening. We see that using low σ values for test data sharpening causes gradual deterioration of accuracy as the α value, i.e., the scaling factor, increases.

As previously mentioned, the success of our approach depends on the selection of the ideal (σ, α) values for test data sharpening, and this depends strongly on how much the validation data is representative of test data. As such, one strategy of finding a better (σ, α) combination is by procuring sufficient amount of validation data. Recall that we did this for OPPORTUNITY dataset and allocated much more validation data compared to other approaches. Another strategy for selecting better parameter values is choosing effective value ranges and intervals for σ and α parameters. Defining smaller value intervals is better than defining large intervals for both σ and α, and the value ranges of the two parameters should include the peak accuracy to exhaustively cover the promising parameter value candidates. Most importantly, exploiting the divide and conquer approach, whenever possible, will much aid in better selecting the effective (σ, α) values since adjusting the value that works for many activity classes is more difficult than adjusting the value for few classes.

Although deep learning models prefer large data, in this paper we returned to the basics and carefully analyzed the *quality* of data. Recall that in the case of OPPORTUNITY dataset, only a small number of sensors (i.e., the lower body sensors) were selected in our experiments, and the train data for learning activity models were also selectively used (i.e., only ADL data and no drill data were used). Even though more features generally add more information to the model, and more data provide more cases for learning a better HAR model, we chose only those features and data that we thought were relevant and of safe quality, and this strategy paid off.

The main advantage of our approach is that the candidate (σ, α) combinations, which can increase the activity recognition accuracy, are many. We were able to confirm this in Figure 12; the recognition accuracy plateaus were formed above the baseline (no sharpening) model across many σ and α values. On the other hand, the shortcoming of our approach is that if the HAR performance of the validation data is saturated, i.e., close to 100%, then the selection of useful (σ, α) becomes difficult. This occurred during the experiments with UCI HAR dataset, and we troubleshooted the situation by splitting the test data in half. Another limitation is that finding the correct (σ, α) becomes difficult if the validation data is not representative of test data. To tackle this problem, we plan to investigate the effect of selectively sharpening the partial features of test data as opposed to sharpening of the entire features of test data that we did in this study.

### 5.2. Model Complexity

The proposed two-stage HAR requires a minimum of three activity recognition models, which generally causes the overall model complexity to increase. For example, in the case of the three models built on the lower body OPPORTUNITY dataset (Figure 8 and Figure 9) for classifying the four activity classes, a total of 1,126,846 CNN parameters were trained. That is, 525,042 parameters for training abstract activity model and 300,902 and 300,902 parameters for training UP and DOWN activity model respectively. In contrast, an end-to-end single 1D CNN model that classifies the same four activities using the lower body OPPORTUNITY dataset required 528,004 CNN parameters to be trained (Figure 15 End to End (4-class), 1D CNN, red bar). The proposed two-stage model’s complexity was more than double the end-to-end model. The recognition accuracy of the two-stage model was 94.27% while the accuracy of the end-to-end model was 93.27%. In general, the complexity of the two-stage model is at a disadvantage to the end-to-end model, but by replacing part of the two-stage model with other simpler models, for example, in the case of Figure 15, replacing the DOWN activity 1D CNN model with a logistic regression classifier (Figure 15 Down Position, Logistic Regression outputs 100% accuracy), we can reduce the complexity of the overall two-stage model. Such a strategy can be actively employed to reduce the model complexity of the two-stage models.

## 6. Conclusions

We presented a divide and conquer approach for 1D CNN-based HAR using test data sharpening for improving HAR performance. We formulated a two-stage HAR process by identifying abstract activities using a confusion matrix. A simple test data sharpening method using Gaussian filter generated a broad range of possible activity recognition accuracy improvements. Our divide and conquer 1D CNN approach was meaningful in both building a better HAR model and selecting useful (σ, α) value for effective test data sharpening. Our method is simple and effective, and is easy to implement once abstract activities suitable for the first-stage can be identified. In the future, we plan to investigate feature-wise sharpening of test data and its effect on asymmetric validation and test data.

## Figures and Tables

**Table 1:** Activity class constitution and number of samples in OPPORTUNITY dataset; the same class label constitution applied to both the lower and upper body sensor data.

Class Division	Up		Down		Total
	Stand	Walk	Sit	Lie	
Train	13,250	7403	6874	1411	28,938
Validate	5964	3216	3766	663	13,609
Test	5326	3885	3460	793	13,464

**Table 2:** Activity class constitution and number of samples in UCI HAR dataset.

Class Division	Dynamic			Static			Total
	Walking	WU^*^	WD^*^	Sitting	Standing	Laying	
Train	1226	1073	986	1286	1374	1407	7352
Test	496	471	420	491	532	537	2947

WU*: Walking Upstairs, WD*: Walking Downstairs.

**Table 3:** Test data sharpening parameter values used in evaluation experiments.

Dataset	OPPORTUNITY		UCI HAR			
Model	Up (lower body)	Up (upper body)	Dynamic1	Dynamic2	Static1	Static2
σ	3	3	8	7	5	8
α	13	12	2.40	1.95	0.06	0.07

**Table 4:** $F_{1}$ score comparison with state of the art approach on lower body OPPORTUNITY dataset. The number in bold indicates the highest $F_{1}$ score.

Dataset	Ordóñez and Roggen [28] (%)		Ours (%)
	Baseline CNN	DeepConvLSTM	CNN+Sharpen
OPPORTUNITY	91.2	93.0	**94.2**

**Table 5:** Confusion matrix of our CNN+Sharpen approach using lower body OPPORTUNITY dataset. The bold numbers in diagonal indicate correctly classified instances; the bottom right bold number indicates the overall accuracy.

		Predicted Class				Recall (%)
		Stand	Walk	Sit${}^{*}$	Lie${}^{*}$	
**Actual Class**	Stand	**5,210**	116	0	0	97.82
	Walk	655	**3,230**	0	0	83.14
	Sit${}^{*}$	0	0	**3,460**	0	100.00
	Lie${}^{*}$	0	0	0	**793**	100.00
**Precision (%)**		88.83	96.53	100.00	100.00	**94.27**

* Test data enhancement was NOT applied to these classes.

**Table 6:** Comparison with the state of the art approaches using UCI HAR dataset. The number in bold indicates the highest accuracy.

Approaches	Accuracy (%)
SVM [10]	96.37
DCNN+ [12]	97.59
FFT+Convnet [14]	95.75
TSCHMM [13]	96.74
CNN+Sharpen (Ours)	**97.62**

**Table 7:** Confusion matrix of our CNN+Sharpen approach on UCI HAR dataset. The bold numbers in diagonal indicate correctly classified instances; the bottom right bold number indicates the overall accuracy.

		Predicted Class						Recall (%)
		Walk	WU	WD	Sit	Stand	Lay	
**Actual Class**	Walk	**491**	2	3	0	0	0	98.99
	WU	3	**464**	4	0	0	0	98.51
	WD	1	5	**414**	0	0	0	98.57
	Sit	0	0	0	**454**	37	0	92.46
	Stand	0	0	0	14	**518**	0	97.37
	Lay	0	0	0	1	0	**536**	99.81
**Precision (%)**		99.19	98.51	98.34	96.80	93.33	100.00	**97.62**

**Table 8:** Comparison of activity recognition accuracy without (1D CNN only) and with test data sharpening (1D CNN+Sharpen). The bold numbers indicate the highest accuracy for each model.

Dataset (Model)	Class	1D CNN Only (%)	1D CNN + Sharpen (%)
OPPORTUNITY (Up)	2	91.28	**91.63**
UCI HAR (Dynamic)	3	97.98	**98.70**
UCI HAR (Static)	3	96.60	**96.67**
